# Effect of Artificial Intelligence Helpfulness and Uncertainty on Cognitive Interactions with Pharmacists: Randomized Controlled Trial

**DOI:** 10.2196/59946

**Published:** 2025-01-31

**Authors:** Chuan-Ching Tsai, Jin Yong Kim, Qiyuan Chen, Brigid Rowell, X Jessie Yang, Raed Kontar, Megan Whitaker, Corey Lester

**Affiliations:** 1 Department of Clinical Pharmacy College of Pharmacy University of Michigan Ann Arbor, MI United States; 2 Department of Industrial and Operations Engineering College of Engineering University of Michigan Ann Arbor, MI United States

**Keywords:** CDSS, eye-tracking, medication verification, uncertainty visualization, AI helpfulness and accuracy, artificial intelligence, cognitive interactions, clinical decision support system, cognition, pharmacists, medication, interaction, decision-making, cognitive processing

## Abstract

**Background:**

Clinical decision support systems leveraging artificial intelligence (AI) are increasingly integrated into health care practices, including pharmacy medication verification. Communicating uncertainty in an AI prediction is viewed as an important mechanism for boosting human collaboration and trust. Yet, little is known about the effects on human cognition as a result of interacting with such types of AI advice.

**Objective:**

This study aimed to evaluate the cognitive interaction patterns of pharmacists during medication product verification when using an AI prototype. Moreover, we examine the impact of AI’s assistance, both helpful and unhelpful, and the communication of uncertainty of AI-generated results on pharmacists’ cognitive interaction with the prototype.

**Methods:**

In a randomized controlled trial, 30 pharmacists from professional networks each performed 200 medication verification tasks while their eye movements were recorded using an online eye tracker. Participants completed 100 verifications without AI assistance and 100 with AI assistance (either with black box help without uncertainty information or uncertainty-aware help, which displays AI uncertainty). Fixation patterns (first and last areas fixated, number of fixations, fixation duration, and dwell times) were analyzed in relation to AI help type and helpfulness.

**Results:**

Pharmacists shifted 19%-26% of their total fixations to AI-generated regions when these were available, suggesting the integration of AI advice in decision-making. AI assistance did not reduce the number of fixations on fill images, which remained the primary focus area. Unhelpful AI advice led to longer dwell times on reference and fill images, indicating increased cognitive processing. Displaying AI uncertainty led to longer cognitive processing times as measured by dwell times in original images.

**Conclusions:**

Unhelpful AI increases cognitive processing time in the original images. Transparency in AI is needed in “black box” systems, but showing more information can add a cognitive burden. Therefore, the communication of uncertainty should be optimized and integrated into clinical workflows using user-centered design to avoid increasing cognitive load or impeding clinicians’ original workflow.

**Trial Registration:**

ClinicalTrials.gov NCT06795477; https://clinicaltrials.gov/study/NCT06795477

## Introduction

Clinical decision support systems (CDSS) are tools that use medical knowledge and health information to aid clinicians’ decision-making to provide enhanced patient care [1,2]. CDSS can be classified into 2 types: knowledge-based and non–knowledge-based [2]. In knowledge-based CDSS, relevant information is evaluated by a set of IF-THEN rules, and recommendations are generated. Non–knowledge-based CDSS use artificial intelligence (AI) and machine learning (ML) methods rather than rules to evaluate information and generate recommendations [3]. Various CDSS have been designed to aid pharmacists’ clinical work, including checking for drug-drug interactions [4], antibiotics stewardship [5], and drug utilization review [6].

Pharmacists’ medication product verification is an example of a pivotal yet time-consuming and vigilant process. During medication product verification, pharmacists compare the contents of a filled medication bottle to professional, close-up images of medication that are integrated into the dispensing platform. Although most pharmacists are checking a physical bottle with the pills inside, the industry is moving toward verification using images of filled medication bottles. Pharmacists spend 30%-48% of their time verifying and dispensing medications [7,8]. Multiplying the time by the mean salary of a pharmacist [9], this equals US $38,823-US $62,117 per pharmacist per year. While some states in the United States have implemented a technician final product verification (tech-check-tech), the cost is still significant as the mean annual wage for a pharmacy technician in 2023 was US $43,330.[10] Furthermore, the tech-check-tech workflow does not eliminate the potential for human error.

Product verification is not only time-consuming and costly, but it also requires pharmacists to be vigilant, which can cause fatigue, cognitive overload, and even result in verification and dispensing errors. Medication dispensing error can be defined as “any deviations of the prescription order,” including but not limited to dispensing the wrong dosage, strength, dose form, or pharmaceutical ingredient [11]. The estimated rate of medication dispensing errors is 2.4 in 100 prescriptions [12] in community pharmacies, with the most common types being wrong ingredients, wrong strength, and labeling errors [12,13]. These errors can result in unfavorable therapeutic outcomes, which can have adverse effects on patient safety and outcomes.

Leveraging its capability to process large amounts of data, AI-based CDSS can help pharmacists reduce cognitive load and maintain vigilance. The term vigilance is used to describe the level of maintaining focus and alertness over a prolonged period of time [14]. Human vigilance tends to decrease over time, and multiple theories explain the phenomenon, including the overload theory (reduction of information-processing abilities), underload theory (mindlessness due to repetitive nature), and fatigue [15-17]. Since AI has the power to process large amounts of information, it can reduce humans’ cognitive load and maintain their information-processing ability. However, balancing transparency and information load is necessary to avoid cognitive overload. Providing too much information can come at the expense of making the task more cognitively demanding. Marusich et al [18] found increasing information in safety-critical situations decreased users’ situational awareness and performance. Similarly, Wu et al [19] examination of high, moderate, and low-complexity user interfaces found significant differences between high- and low-complexity interfaces. Low-complexity interfaces significantly increased users’ attention and decreased cognitive load compared with high-complexity interfaces (*P*<.05). Furthermore, cognitive load is not static. Experts and novices at a given task have different levels of cognitive overload; expert users have significantly less cognitive load than novices when using an interface of the same level of complexity [19]. Therefore, it is critical to meet the information needs of users in the design of AI advice.

AI and ML–based CDSS are often “black box” systems, where there is a lack of insight into the AI and ML prediction. As a result, there is an increasing call for greater AI transparency among medical professionals, emphasizing the need to display the uncertainty of AI-generated results [20,21]. While presenting this uncertainty may initially confuse users and require more cognitive effort to make decisions [22], it has significant benefits. Displaying uncertainty helps mitigate issues with AI and ML like overreliance and bias, which fosters better human-AI collaboration and trust [22,23]. This transparency enables users to recognize that AI and ML predictions are not definitive, encouraging them to incorporate their judgment into decision-making. In addition, showing uncertainty serves as a new stimulus that maintains user vigilance, preventing boredom or mindlessness as suggested by the underload theory [17].

AI and ML–based CDSS have the potential to improve medical care if the systems perform well and are appropriately implemented [24]. However, when the AI provides inaccurate, unreliable, and biased results, it can lead users to decision errors due to mechanisms such as automation bias and algorithmic aversion [25]. Automation bias is defined as people’s heuristic belief that automation’s performance is consistent when the system’s performance is not always perfect, which can lead to users accepting incorrect advice or rejecting correct advice [25]. Conversely, algorithmic aversion reflects the tendency of users to not trust the algorithm’s performance after seeing it make mistakes, even when its performance is better than humans [26]. In a study of users’ assessments of AI and human errors in high-stakes situations (ie, medical and legal), users rated AI errors significantly more harshly and less fair than human errors (*P*<.001) [25]. This result was echoed by Dietvorst et al [26], who studied the effect of AI errors on participants’ correctly predicting outcomes. Despite the AI significantly outperforming the humans, participants were significantly less likely to trust the AI’s prediction and their confidence in the AI significantly decreased after seeing an AI error. To prevent such human errors in clinical decision-making with the help of AI, high-performing and reliable systems are pivotal. In this study, we assess the impact of helpful and unhelpful AI on user cognitive workload.

Previously, our group developed an AI prototype aiming to help pharmacists with medication product verification and reduce dispensing errors. Using the participatory design method, researchers conducted focus groups with pharmacists who had medication verification experience to understand the workflow, difficulties, and concerns of the current process, and ideas about incorporating AI to aid this process. Based on pharmacists’ feedback, a user-centered AI interface emphasizing clarity and accessibility was developed. The development of the AI help software is detailed in previous research by Zheng et al [27]. The purpose of this paper is to establish how pharmacists cognitively incorporate and use an AI prototype during the medication product verification process by analyzing eye-tracking fixation data.

## Methods

### Participants and Trial Design

This study was a randomized controlled trial. We recruited 30 pharmacists to participate in a single, remote study visit. Out of 2 listservs for professional pharmacists, the Minnesota Pharmacy Practice-Based Research Network and the University of Michigan College of Pharmacy Preceptor Network were used to recruit participants. The listserv managers sent a recruitment email instructing interested pharmacists to contact the study team to schedule a screening phone call. The study’s inclusion criteria were (1) a licensed pharmacist in the United States, (2) at least 18 years old at the time of screening, and (3) access to a laptop or desktop computer with a webcam to complete the experiment. The exclusion criteria were (1) require assistive technology to use the computer, (2) need eyeglasses with more than 1 power (eg, bifocals) to complete the experiment, (3) have uncorrected cataracts, intraocular implants, glaucoma, or permanently dilated pupils, or (4) eye movement or alignment abnormalities. A random number generator created in R (R Core Team) assigned each participant to receive black box or uncertainty-aware help. The probability of being assigned to black box help trials or uncertainty-aware help trials was equal for all participants as was the probability of completing the first 100 trials with AI help or without AI help. In each trial, participants would check the images on the screen and decide whether it was a good fill (the medication in the fill image is identical to the medication in the reference image) or a bad fill (the medication in the fill image and the reference image are different) and hit either the reject or the accept button.

To ensure confidence in the quality of the remote data collection, all participants met online with a researcher before performing any study activities. The purpose of the online meeting was to ensure the study setup was consistent (eg, adequate lighting and no glare) and participants had the required materials (ie, ruler or tape measure, and standard ID) to conform with Labvanced’s eye-tracking and calibration requirements. After meeting with the researcher, all participants watched an orientation video which provided a detailed overview of the study including an explanation of how the AI software makes its predictions and an introduction on how to use the interface to perform the verifications.

### The Task: Pharmacists’ Medication Verification

#### Overview

**Figure 1:**
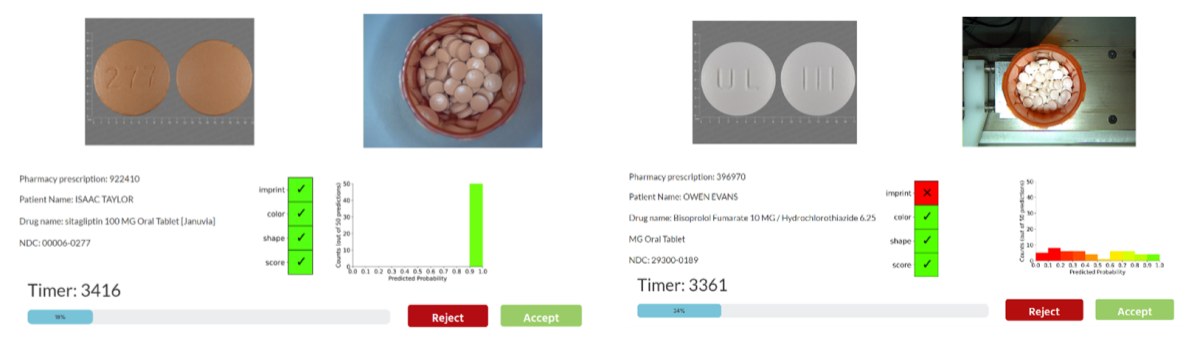
The medication verification system interface with AI Help. In trials without AI assistance, pharmacists only have access to the reference image (refImage) and fill image. With black box AI help, pharmacists saw an AI match plot, which shows the AI’s stance on the match status for 4 key medication characteristics (imprint, color, shape, and score). In uncertainty-aware AI help, in addition to the match plot, pharmacists also saw the AI histogram, which displays the probability distribution from 50 predictions, indicating the uncertainty of the prediction. Examples of high certainty (left-hand image) and low certainty (right-hand image) are shown. AI: artificial intelligence.

Pharmacists are responsible for dispensing the correct medication to patients to ensure optimal therapeutic outcomes. Before dispensing medications to patients, pharmacists must visually inspect the medication being filled and compare them to the prescribed drugs. This process is called medication product verification. In the study, each participant performed 200 medication product verification tasks, where their eye movement was recorded using an eye tracker. Each participant conducted 100 medication verifications without AI assistance and 100 verifications according to their assigned AI help group. A screenshot of the participants’ view is in Figure 1. To avoid the carry-over effect, the medications being verified in no AI help trials were different from those with AI help. However, the medications were identical in trials with black box AI help and trials with uncertainty-aware AI help.

#### Trial Type

Each trial was configured with specific variables, including type of AI assistance, case type, and participant performance. There were 3 categories of AI assistance (1) no help, (2) black box help, and (3) uncertainty-aware help. In all 3 categories, 76 of the 100 (76%) trials displayed correctly filled medications. In the no-help condition, participants used only the reference image and filled the image and text. Black box help added an AI-generated match plot, while uncertainty-aware help also included an AI-generated probability histogram. The case type, relevant only when AI assistance was provided, referred to the alignment between the AI’s recommendation and the correct answer. The AI accurately recommended the correct action (accept or reject) 79% of the time. There were 2 scenarios: (1) helpful advice, where the AI’s guidance matched the correct action (accept or reject), and (2) unhelpful advice, where the AI’s suggestion opposed the correct answer.

#### The AI Help Software

We previously developed an automated tool using a Bayesian neural network [28] to predict the National Drug Code (NDC), color, and shape of medications using images of pills in a prescription bottle [29]. In this study, we modified the Bayesian model [30] to predict the dispensed pills’ NDC and the associated model’s uncertainty of the predictions. This was accomplished by applying the random dropout technique [30] to the ResNet-34 [31] convolutional neural network. The dropout technique is realized by a layer in the neural network called the dropout layer, which activates connections in the layer with certain probabilities. As such, the output of the layer is random every time it predicts, which we use to measure the uncertainty of the prediction. In our research, the model generated 50 potential probabilities for every image. Because the distribution of predicted probabilities could not be written mathematically in close forms, it was approximated using samples drawn from the distribution.

A sample size of 50 was used to balance between computational cost and goodness of approximation. The AI software then compares the predicted NDC and the reference image NDC to generate insights on the match status. The final interface had 2 AI-generated regions that were designed to aid pharmacists’ medication verification the AI match plot and AI histogram. The AI match plot showed the match status of 4 characteristics between the AI-predicted NDC and the expected NDC on the prescription which were (1) imprint, (2) color, (3) shape, and (4) score. The unmatched characteristics were denoted by a red “X,” while matched ones by a green check. Figure 1 shows a prediction with 4 green checks.

The AI histogram displayed the probability distribution from 50 AI predictions, each representing the AI’s estimated likelihood that its predicted NDC matches the expected NDC. These probabilities form the histogram, illustrating the AI software “confidence” in the match status. A green peak in the histogram indicates consistent results across the 50 simulations, suggesting high confidence. Conversely, a flat, colorful distribution signifies low confidence. In Figure 1, the left-hand image shows a highly confident prediction and the right-hand image shows a low confidence prediction.

A crucial component of this study was to examine how pharmacists interact with AI-generated errors. The model’s overall accuracy is 98.46%. For the purpose of this study, errors were oversampled to obtain a sufficient number of mistakes which lowered the model’s accuracy to 79% for the experiment. At 79% accuracy, our model is above the 0.70 reliability threshold wherein imperfect automation is preferable to no automation [32]. Lowering the accuracy balanced the number of trials and participants’ time commitment to the study.

#### Eye-Tracking Data

Eye-tracking data were collected using software from Labvanced (Paderborn). Labvanced uses deep learning models to process webcam videos, allowing online eye movement tracking. The accuracy and precision are comparable to in-laboratory “gold standard” eye trackers and are verified in a peer-reviewed paper [33]. Participants logged on to the Labvanced Trials website. First, they verified the webcam accurately captured their face and agreed to the recording and data use policy before starting the trial. Participants were then prompted to complete a demographics questionnaire that included questions such as age, gender, race, pharmacy practice experience level, practice setting, and trust level in automated systems.

The first phase of the trial was the calibration. During calibration, participants were first asked to measure the distance of their face to the screen and set the center pose that worked best. The actual calibration included 2 parts: (1) the position and orientation and (2) fixation (ie, focused gaze) to certain points on the screen. Participants were asked to follow and fixate on 2 series of red dots on the screen while maintaining their faces at the center pose. After the calibration was completed, participants started the medication verification trials. The system continuously monitored participants’ facial position and orientation. If participants moved out of the center pose, they were prompted to re-align their face to the center pose and rerun the fixation calibration. The raw eye-tracking data were processed by the Labvanced algorithm [34] to fixation data. Our analyses were based on the fixation data and excluded eye movement known as saccades, where participants were simply transitioning from 1 fixation to the next and not necessarily fixating in the region.

### Data Analysis and Preprocessing

Fixation analysis is the most common metric for analyzing eye-tracking data. The fixation rate, fixation duration, and dwell time have been proposed to reflect human cognitive interest in certain areas of interest [35-37]. Higher fixation rates and longer dwell times indicate repeated interest in a certain area. The longer fixation duration indicates a higher cognitive load. To characterize the use pattern, we were also interested in the order in which pharmacists use these images. Thus, we report the first fixation region, the last fixation region, the number of fixations in each region, the average fixation duration of each fixation, and the dwell time, which was calculated by the sum of the duration of all fixations in each region in each trial. We further stratified the data based on different AI help types (ie, black box versus uncertainty-aware help), and case types (helpful versus unhelpful advice).

To eliminate calibration failure and measurement error, we calculated the modified *z* score of each region’s dwell time in each trial. The modified *z* score method is more robust in detecting outliers since it uses median instead of mean in the calculation of *z* scores. If the modified *z* score of the dwell time in that region is greater than 3.5, we labeled that observation as an outlier and excluded it from our analyses.

**Figure 2:**
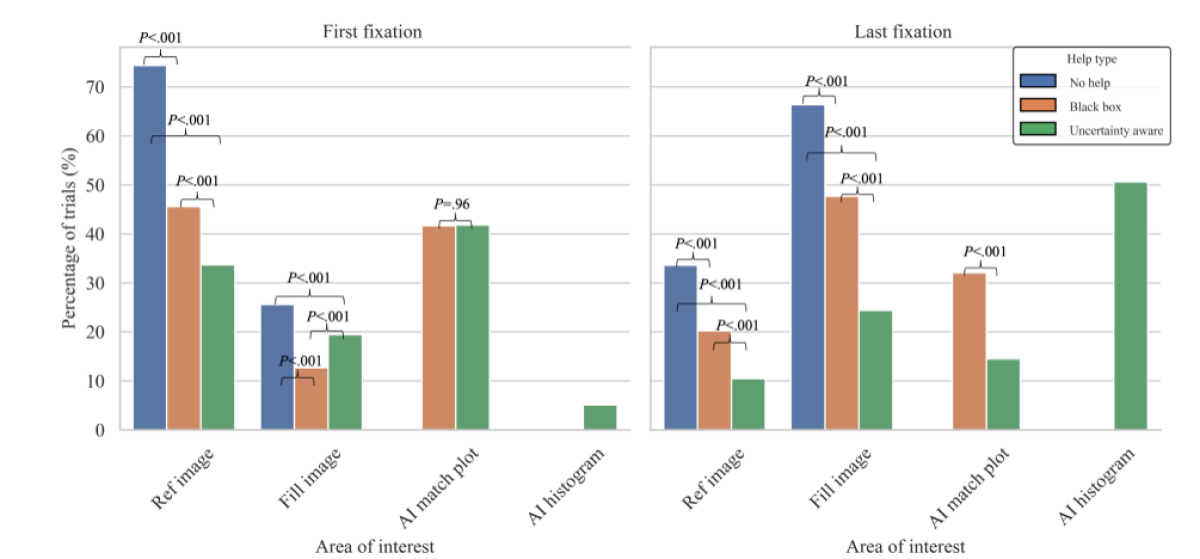
Percentage of trials by region of interest and AI help type. Left: first fixation; right: last fixation. *P* values were calculated for the 2-tailed z tests to determine the significance of the observed differences and are displayed above respective brackets. AI histogram was only present in the uncertainty-aware condition. No comparative statistics exist in this case. AI: artificial intelligence.

For all help type pairs, 2-tailed *z* tests were run for the first and last fixation area to determine if differences among categories were statistically significant (Figure 2). *P* values were also calculated to show if the proportions being compared were statistically significant. Statistical significance between help types was calculated for fixation duration, number of durations, time of fixations per area, and total dwell time by case type using the Mann-Whitney *U* test with the *P* values adjusted using the Bonferroni correction.

### Ethical Considerations

This study did not meet the National Institutes of Health’s definition of a clinical trial and the University of Michigan’s Institutional Review Board determined this research is exempt from institutional review board oversight under federal Exemption 3(i)(A) and/or 3(i)(B) at 45 CFR 46.104(d).

## Results

### Overview

In total, 31 participants were randomized to receive black box or uncertainty-aware AI help. One participant experienced technical difficulties and was unable to complete any trials. Fifteen subjects in each condition completed the experiment and were included in the final analysis. Demographic variables of age, gender, race, practice settings, and working years were well-balanced (*P*>.05). Full details of demographic variables are reported elsewhere [38]. After outlier removal, the dataset comprises 2449 trials without AI assistance, 1365 with black box AI help, and 1391 with uncertainty-aware AI help.

The effects of AI assistance on pharmacists’ decision-making and reaction time are reported in full elsewhere [38]. In summary, performance and reaction times varied by AI type and AI accuracy. In general, uncertainty-aware AI led to faster decision-making and protected against bad AI advice to approve a misfiled medication whereas black box AI increased reaction times and bad AI advice resulted in lower accuracy.

### First and Last Fixation Area

#### Overview

In trials without AI help, the first fixation area (Figure 2, left) was either (1) the reference image (1822/2449, 74.4%) or (2) the fill image (627/2449, 25.6%). In the black box help condition, the first fixation area was either (1) the reference image (622/1365, 45.6%), (2) the fill image (174/1365, 12.7%), or (3) the AI match plot (569/1365, 41.7%). In uncertainty-aware help, it was (1) the reference image (469/1391, 33.7%), (2) the fill image (270/1391, 19.4%), (3) the AI match plot (581/1391, 41.8%), and (4) the AI histogram (71/1391, 5.1%). For trials without AI help, the last fixation area (Figure 2, right) was either (1) the reference image (822/2449, 33.6%) or (2) the fill image (1627/2449, 66.4%). In the black box help condition, it was (1) the reference image (276/1365, 20.2%), (2) the fill image (651/1365, 47.7%), and (3) the AI match plot (438/1365, 32.1%). In uncertainty-aware help, it was (1) the reference image (146/1391, 10.5%), (2) the fill image (339/1391, 24.4%), (3) the AI match plot (202/1391, 14.5%), and (4) the AI histogram (704/1391, 50.6%). All of these comparisons, with one exception, showed statistically significant differences between help types. The *P* values were calculated to be *P<*.05 (*z* score>1.96 or <–1.96), thus rejecting the null hypothesis and suggesting there is a significant difference in proportions between each 2. The one exception to this was the pair for first fixation being AI match plot with black box help versus uncertainty-aware help, where the *z* test was –.45 and the *P* value was .97, suggesting that these values were not statistically significantly different.

#### Number of Fixations

**Figure 3:**
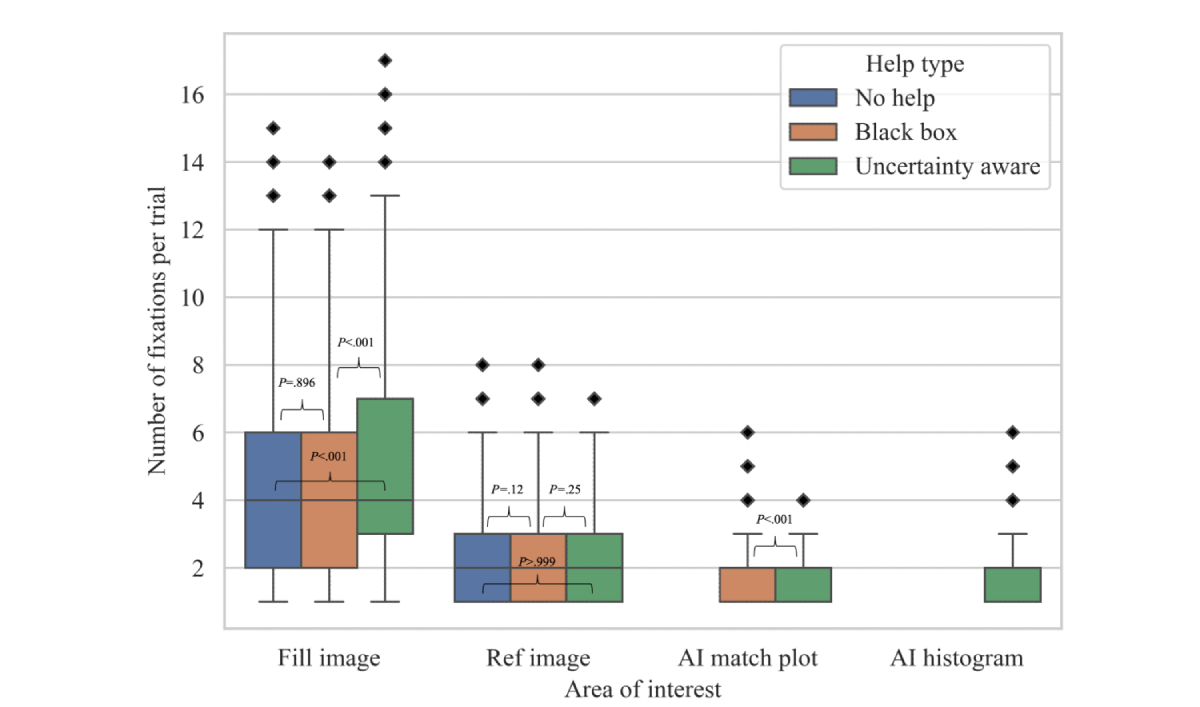
Boxplot representation of the number of fixations in each area per trial, categorized by help type. Bonferroni adjusted *P* values were calculated alongside Mann-Whitney *U* tests, and these statistics are displayed above respective brackets. AI histogram was only present in the uncertainty-aware condition. No comparative statistics exist in this case. AI: artificial intelligence.

Irrespective of help type, fill images had the highest number of fixations in a trial, and the number of fixations per trial did not decrease after the introduction of AI-generated areas (number of fixations: mean [SD], median [IQR]): (1) no help, mean 4.44 (SD 2.92), median 4.0 (IQR 2.0-6.0); (2) black box help, mean 4.33 (SD 2.90), median 4.0 (IQR 2.0-6.0); and (3) uncertainty-aware help, mean 5.14 (SD 3.43), median 4.0 (IQR 3.0-7.0). Reference images received fewer fixations, with the averages and range being (1) no help: mean 2.47 (SD 1.4), median 2.0 (IQR 1.0-3.0), (2) simple help mean 2.38 (SD 1.4), median 2.0 (IQR 1.0-3.0), and (3) advanced help mean 2.45 (SD 1.4), median 2.0 (IQR 1.0-3.0). The AI match plot and AI histogram received a lower number of fixations. The mean (SD) and median (IQR) for the number of fixations in AI match plots in black box and uncertainty-aware help trials are mean 1.91 (SD 1.12), median 2.0 (IRQ 1.0-2.0), and mean 1.65 (SD 0.82), median 1.0 (IQR 1.0-2.0), and in AI histogram in uncertainty-aware help trials, they are mean 1.69 (SD 0.87), median 1.0 (IQR 1.0-2.0), respectively. The numbers are visualized in a box plot in Figure 3. Bonferroni adjusted *P* values comparing no help with black box for fill image and all 3 comparisons for reference image were observed to be greater than .05. Therefore, we do not reject the null hypothesis of the groups being statistically similar. The comparisons of no help and uncertainty-aware and black box with uncertainty-aware advice for fill image, and black box with uncertainty-aware for AI match plot showed significance (Mann-Whitney *U*=1088528.5, 507726.0, and 381013.0, respectively; all *P<*.05).

#### Fixation Duration

**Figure 4:**
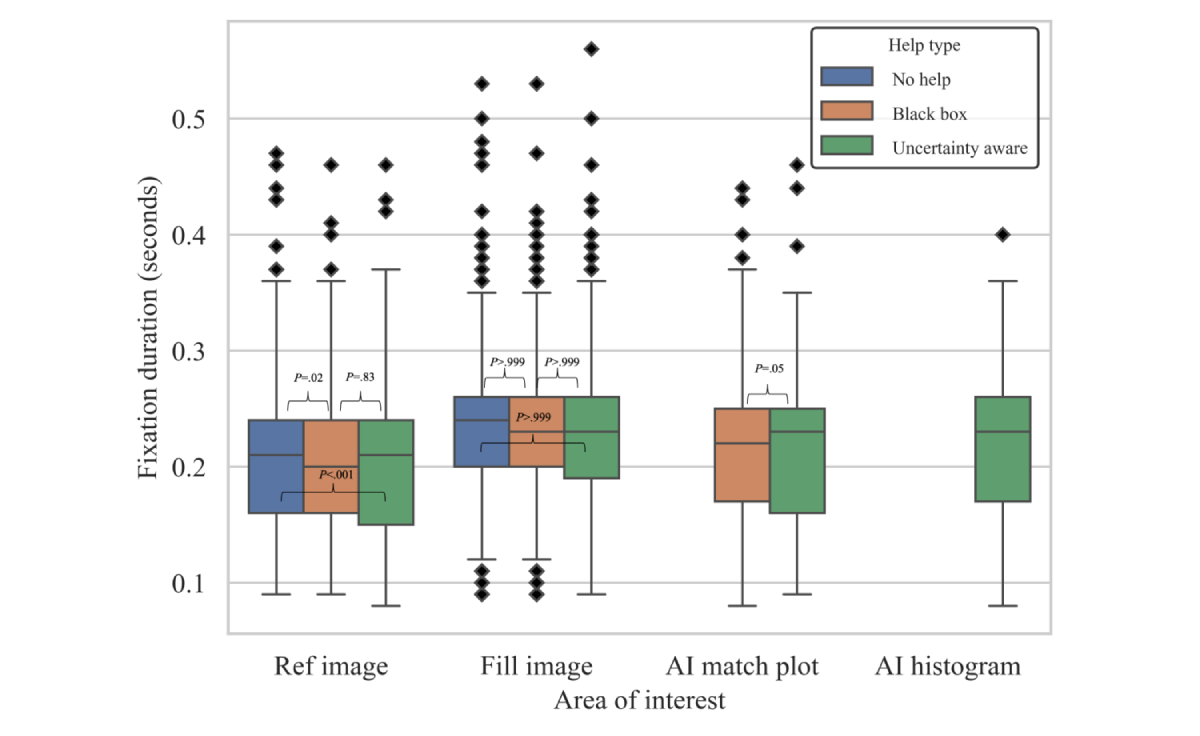
Boxplot representation of fixation duration in each area, categorized by help type. Bonferroni adjusted *P* values were calculated alongside Mann-Whitney *U* tests, with a Bonferroni correction, and these statistics are displayed above respective brackets. AI histogram was only present in the uncertainty-aware condition. No comparative statistics exist in this case. AI: artificial intelligence.

The fixation duration in each region was similar, regardless of help type. The median duration of fixation was around 0.22 seconds, with an IQR of approximately 0.75 seconds. This indicates that the cognitive load for processing information in each area was similar. The boxplot of the finding is in Figure 4. One adjusted *P* value was below .05, with a *U* value of 29067652, comparing no help to uncertainty-aware advice for the fill image. This indicates a meaningful statistical difference between these 2 groups.

#### Dwell Times

**Figure 5:**
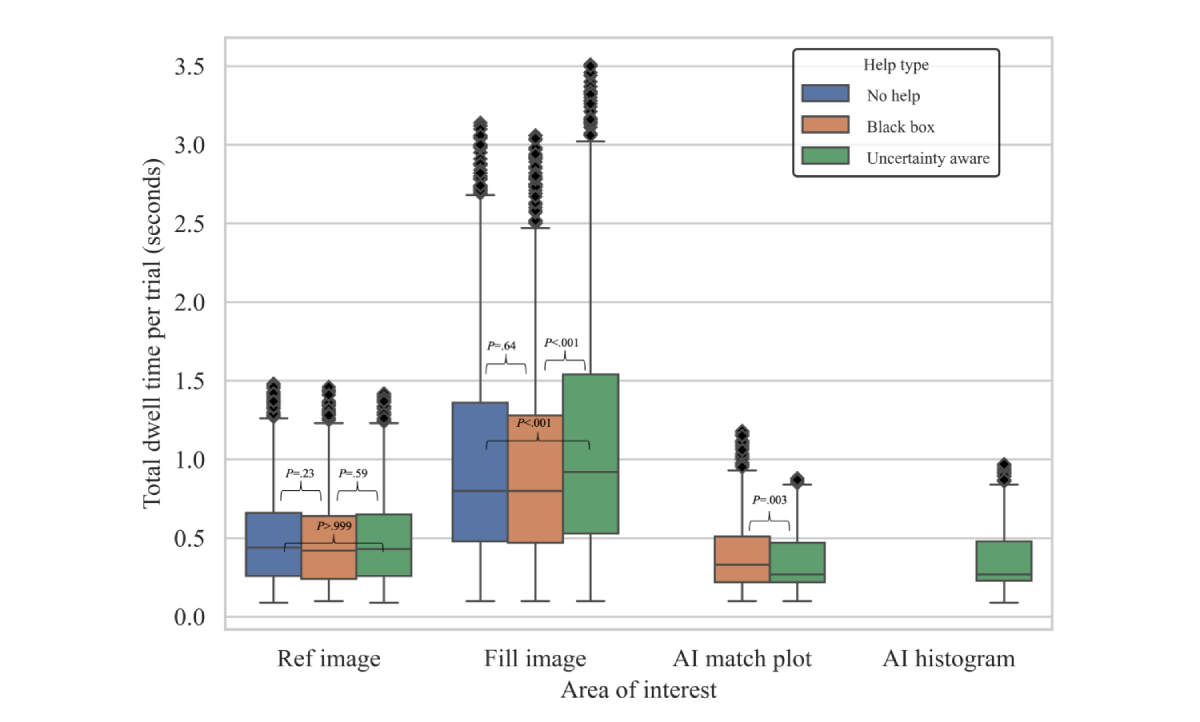
Boxplot representation of dwell time in each area, categorized by help type. Bonferroni adjusted *P* values were calculated alongside Mann-Whitney *U* tests, with a Bonferroni correction, and these statistics are displayed above respective brackets. AI histogram was only present in the uncertainty-aware condition. No comparative statistics exist in this case. AI: artificial intelligence.

Participants consistently spent the most time on fill images. The median (IQR) fill image dwell times (in seconds) were: (1) no help trials, median 0.80 (IQR 0.48-1.36), (2) black box help, median 0.80 (IQR 0.47-1.26), and (3) uncertainty-aware help, median 0.92 (IQR 0.53-1.54). For the reference image, the median (IQR) dwell times (in seconds) were (1) no-help, median, 0.44 (0.26-0.66), (2) black box help, median 0.42 (0.24-0.64), and (3) uncertainty-aware help, median 0.43 (IQR 0.26-0.65). The AI match plots had median (IQR) dwell times of 0.33 (IQR 0.22-0.51) and 0.27 (IQR 0.22-0.47) seconds in black box and uncertainty-aware help trials, respectively. The AI histograms had a median (IQR) dwell time of 0.27 (IQR 0.23-0.48) seconds in uncertainty-aware help trials (Figure 5).

**Figure 6:**
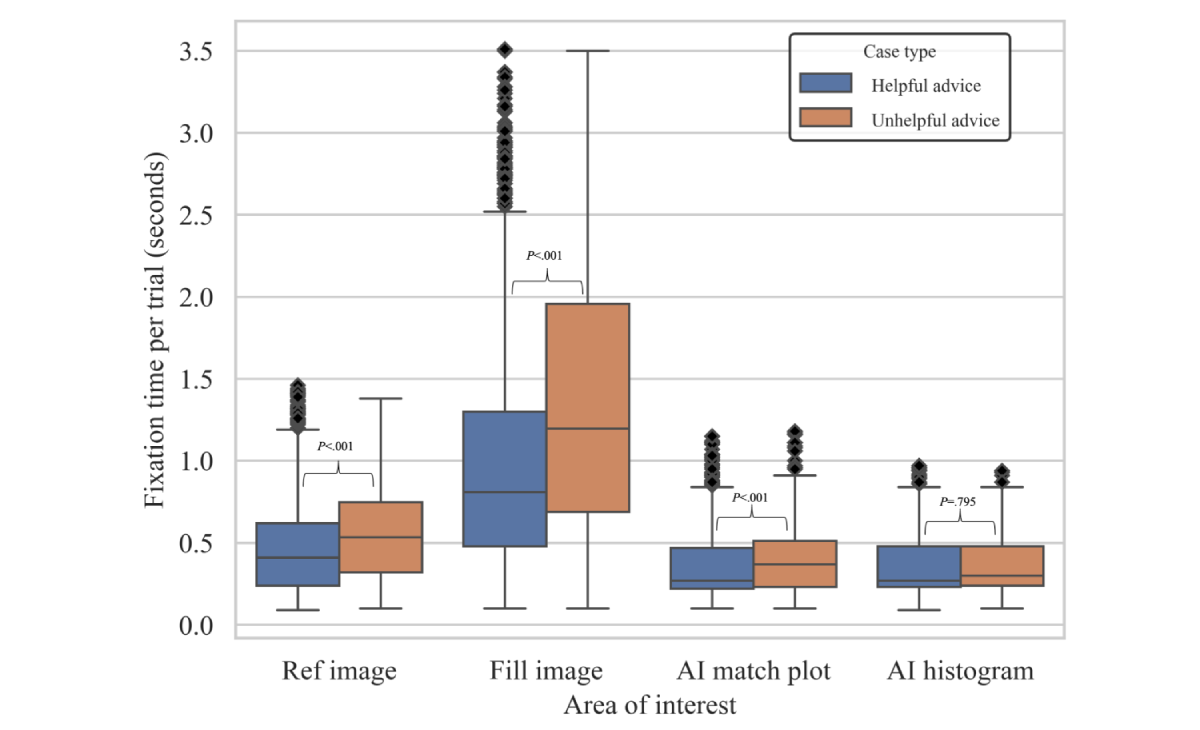
Dwell time in each area by case type. Compared with helpful advice trials, those with unhelpful advice resulted in significantly longer dwell times in fill and reference images. Bonferroni adjusted *P* values were calculated alongside Mann-Whitney *U* tests and these statistics are displayed above respective brackets. AI: artificial intelligence.

The Mann-Whitney *U* test result showed no significant difference between the dwell time in reference images. There was a significant difference in dwell times for fill images with uncertainty-aware help trials compared with no-help trials (*U* value=1113203.0, Bonferroni adjusted *P*<.05) and black box help trials (*U* value=517551.5, Bonferroni adjusted *P*<.05); and for AI match plot from black box to uncertainty-aware (*U* value=375591.0, Bonferroni adjusted *P*<.05). The difference in fill image dwell times was nonsignificant between no help and black box help (Figure 5). Dwell times in fill images and reference images were significantly longer in trials with unhelpful advice compared with helpful advice (the fill image, median: 1.30 vs 0.81 seconds, *U* value 238928.0, *P*<.05; ref image, median: 0.54 vs 0.41 seconds, *U* value 264560.5, *P*<.05; Figure 6).

## Discussion

### Principal Results

Our study presents critical insights into the interaction between pharmacists and an AI-based CDSS. Our findings highlight a shift in the visual and cognitive engagement of pharmacists when AI-driven tools are used. Although communicating the uncertainty of AI has been shown to have benefits such as preventing overreliance and fostering trust in AI [20,22], our study found that it resulted in a longer cognitive processing time of the core task image (ie, filled medication images), though primarily in situations where the AI provided unhelpful advice. The criticality of AI accuracy is underscored by our findings, as AI advice of varying correctness significantly impacts the cognitive processing time of users. These findings suggest the potential of human-AI collaboration to enhance health care delivery if AI is accurate, reliable, and deployed properly without interfering with health care professionals’ existing workflow or increasing their cognitive load.

Irrespective of AI intervention, participants allocated the majority of their fixation time to fill images. The fact that pharmacists need to inspect pills from various angles to verify color, shape, and imprint for accurate identification may give rise to longer cognitive processing times. On the other hand, AI-generated regions have shorter dwell times. This might stem from the graphical simplicity of these images [39] or indicate that users correctly perceive the AI-generated regions as supportive in completing the task. Compared with not showing AI outputs’ uncertainty (ie, black box AI help), we found that displaying uncertainty results in significantly longer dwell times in the original (fill) images, especially when the AI advice was unhelpful. This may suggest that users are confused or second-guess the AI’s correctness when they see the uncertainty histogram, leading to a need to go back to the fill images and verify the correctness of the advice. The shorter dwell times observed with black box AI advice may be attributed to overreliance on automation, resulting in missed errors in the filled medication.

AI-based CDSS influenced participants’ drug verification processes. When participants had access to AI, 19% to 26% of total fixations were shifted to AI regions, and there was a decrease in fixation in the original (fill and reference) images. This indicates that the AI tool changed how participants process information, and participants incorporate AI-generated information in their decision-making process. The decrease in fixation counts in original images and the shift to AI-generated regions indicate that AI may support users in completing the task. When AI help is available, users tend to look at the AI match plot first, and then move on to the fill and reference images. This suggests that providing a simple graphical “summary” of the AI advice may aid users in their decision-making and help them maintain vigilance.

### Comparison With Previous Work

Our finding demonstrates the need for well-designed AI that balances transparency and information load. Since many AI algorithms are essentially a “black box,” there is a demand for AI transparency [23,40]. However, displaying more information about the advice can decrease user vigilance since more information needs to be processed. In designing AI-based systems, there is a need to balance the transparency and explainability of AI output and the amount of information presented to the users. Previous research has shown that high-complexity interfaces significantly increase cognitive workload compared with low-complexity interfaces [19]. Together, our findings and the previous research support the use of our graphically simple, black box AI help over to the histogram which may be overloading the users.

According to Prabhudesai et al [22], users develop their expertise in explaining the uncertainty plot over time, which resolves the initial confusion. In addition to users developing an explanation over time, the user’s expertise with a task impacts the level of cognitive load. Wu et al [19] found at the same level of information load, experts at a task experience significantly less cognitive load than novices. Too much complexity, such as an uncertainty histogram, might not be suitable for the relatively simple medication verification task especially since pharmacists are often tasked with additional responsibilities, which interfere with their vigilance in performing the verification task [41]. Simplifying the communication of the uncertainty might balance the benefits of this kind of advice from AI.

The results revealed that AI’s correctness (ie, helpfulness) had a significant impact on users’ cognitive processing time. Helpful advice resulted in significantly shorter dwell times in reference images and fill images compared with unhelpful advice. This finding supports that users are susceptible to automation bias, the heuristic tendency of users to favor the suggestion made by automated systems [25], and underlines the importance of AI’s correctness and performance. Developers should validate AI’s accuracy and reliability before implementing it in the real world, for the underperformance of AI can introduce new user errors through mechanisms such as automation bias, slowing users down. An important way to do this is through the development of standards and guidelines, as well as assessing accuracy, reliability, and other performance metrics [40]. Park et al [42], introduced a framework for AI-based medical products, emphasizing phases akin to drug trials which are (1) balancing benefits and risks, (2) confirming usability through methods like AB testing, (3) conducting large-scale trials to validate effectiveness, and (4) ongoing monitoring for self-improvement. Like medications, AI-driven CDSS can profoundly impact patient care, necessitating rigorous pre- and post-deployment validation.

How AI and CDSS can be used in medication dispensing is under-researched. A systematic review of CDSS and medication use found that while CDSS is used in the prescribing, administrating, and monitoring phases of medication use, CDSS has not been deployed for medication dispensing [43]. To the best of our knowledge, our innovative study is the first to examine how a CDSS impacts cognitive workload for medication verification and dispensing. Our study adds important insights into supporting pharmacists who are experiencing high levels of burnout. A nationwide survey of community pharmacists found that 74.9% of pharmacists have experienced burnout [44]. Furthermore, high prescription volumes and inadequate pharmacist coverage are contributing factors to dispensing errors [45]. This is concerning as nearly a third (32.1%) of community pharmacists work alone [46], and community pharmacists spend nearly half their work hours (48%) verifying prescriptions [47]. Wash et al [48] narrative review of pharmacists’ well-being and burnout found a lack of research into interventions to assist pharmacists with burnout. AI tools such as ours offer a potential solution to alleviate burnout and prevent dispensing errors by providing a second opinion and decreasing cognitive workload.

The use of user-centered design is becoming increasingly prominent in CDSS development [49-53]. A 2024 review of CDSS highlighted the importance of user-centered design to foster the trustworthiness, usability, and acceptability of CDSS tools by health care providers [54]. CDSS tools should fit into current workflows and address health care providers’ needs and preferences [54]. During the user-centered design phase of developing our AI prototype, pharmacists emphasized the need for simple and accessible information to guide them in completing the task [27]. To avoid overwhelming pharmacists with redundant information, the match plot only displays AI’s stance on color, shape, imprint, and score, which pharmacists identified as the most important information during the medication verification process [27]. Our study adds to the literature a pharmacy-based user-centered design use case underscoring the importance of incorporating user feedback and preferences into the design process. Developers of CDSS should focus on creating systems that align closely with user needs and preferences which can enhance usability and effectiveness. By prioritizing user-centered design principles, developers can improve the performance of AI-based systems and ensure that these systems effectively augment rather than disrupt user workflows.

### Limitations and Future Work

Several other eye-tracking metrics have been proposed to assess cognitive load. Voluntary movements such as saccade length and saccade velocity, and involuntary movements such as pupil dilation can reflect cognitive load. Due to the lack of data availability, we did not include these metrics in our analysis. Future studies can include these eye-tracking metrics to further understand users’ cognitive workload.

This study showcases the need for well-designed AI that balances transparency and information load. Future research should focus on conducting user-center design and usability testing before implementation to understand the benefits and drawbacks of using the AI tool. Also, qualitative methods, such as interviews, and quantitative methods, such as surveys, can incorporated throughout the design and testing phases to allow for design revision based on participants’ perceptions of the AI tools. This may help inform the design and implementation of AI tools in augmenting health care professionals’ workflow. As Wu et al [19] described, the interaction between a user’s level of expertise and the complexity of an AI tool has a significant impact on the user’s cognitive load. Future research should consider allowing users to customize the AI display based on their expertise with the task and comfort with the AI tool. Future studies can include qualitative and quantitative methods to further understand user experiences.

Pharmacists routinely perform a variety of tasks concurrently with the medication verification including checking for drug-drug interactions, confirming the medication count is correct, and verifying patient directions are accurate, complete, and written in patient-friendly language. Pharmacy-specific future research should explore new designs to support dispensing tasks and reduce cognitive load.

### Conclusions

The goal of this study was to assess how pharmacists cognitively incorporate and use an AI prototype during the medication product verification process, using eye-tracking data to analyze their interaction with the system. The study revealed that AI-based CDSS can alter traditional workflow and cognitive engagement, with pharmacists allocating significant focus to AI-generated regions, which indicates the integration of AI advice in decision-making. However, communicating AI uncertainty by displaying a probability histogram increased cognitive processing time for original images. The correctness of AI suggestions directly affects cognitive processing, with helpful AI advice reducing and unhelpful AI advice increasing it. The findings underscore the importance of accurate, reliable AI in health care and suggest that user-centered design and AI transparency are crucial for effective human-AI collaboration. Future research should assess additional eye-tracking metrics to determine users’ cognitive load, incorporate user-centered design to inform the CDSS design, use qualitative and quantitative methods throughout the design and testing phases to better understand users’ experiences, and focus on assisting pharmacists with the varied tasks involved in medication dispensing.

## Appendix

Multimedia Appendix 1 CONSORT-eHEALTH checklist (V 1.6.1).

## Abbreviations

AI: artificial intelligence
CDSS: clinical decision support systems
ML: machine learning
NDC: National Drug Code

## Data Availability

The datasets generated during and/or analyzed during this study are available from the corresponding author on reasonable request.
